# Risk of advanced fibrosis in first-degree relatives of patients with nonalcoholic fatty liver disease

**DOI:** 10.1172/JCI162513

**Published:** 2022-11-01

**Authors:** Nobuharu Tamaki, Noora Ahlholm, Panu K. Luukkonen, Kimmo Porthan, Suzanne R. Sharpton, Veeral Ajmera, Yuko Kono, Shravan Dave, Aijaz Ahmed, Vinay Sundaram, Michael J. Wilkinson, Heather Patton, Hersh Gupta, Vanessa Cervantes, Christie Hernandez, Scarlett J. Lopez, Ria Loomba, Amanda Baumgartner, Lisa Richards, Perttu E.T. Arkkila, Katriina Nemes, Helena Isoniemi, Hannele Yki-Järvinen, Rohit Loomba

**Affiliations:** 1 Nonalcoholic Fatty Liver Disease Research Center, Division of Gastroenterology and Hepatology, Department of Medicine, University of California San Diego, La Jolla, California, USA.; 2 Department of Gastroenterology and Hepatology, Musashino Red Cross Hospital, Tokyo, Japan.; 3 Department of Medicine, University of Helsinki, Helsinki University Hospital, Helsinki, Finland.; 4 Minerva Foundation Institute for Medical Research, Helsinki, Finland.; 5 Department of Internal Medicine, Yale University, New Haven, Connecticut, USA.; 6 Division of Gastroenterology and Hepatology, Department of Medicine, University of California San Diego, La Jolla, California, USA.; 7 Division of Gastroenterology and Hepatology, Stanford University School of Medicine, Stanford, California, USA.; 8 Karsh Division of Gastroenterology and Comprehensive Transplant Center, Cedars-Sinai Medical Center, Los Angeles, California, USA.; 9 Division of Cardiovascular Diseases, Department of Medicine, University of California San Diego, La Jolla, California, USA.; 10 Gastroenterology Section, VA San Diego Healthcare System, San Diego, California, USA.; 11 Department of Gastroenterology and; 12 Transplantation and Liver Surgery Unit, Abdominal Center, University of Helsinki, Helsinki University Hospital, Helsinki, Finland.; 13 Division of Epidemiology, Department of Family Medicine and Public Health, University of California San Diego, La Jolla, California, USA.

**Keywords:** Gastroenterology, Hepatology, Epidemiology, Fibrosis, Liver cancer

## Abstract

**BACKGROUND:**

A pilot, single-center study showed that first-degree relatives of probands with nonalcoholic fatty liver disease (NAFLD) cirrhosis have a high risk of advanced fibrosis. We aimed to validate these findings using 2 independent cohorts from the US and Europe.

**METHODS:**

This prospective study included probands with NAFLD with advanced fibrosis, NAFLD without advanced fibrosis, and non-NAFLD, with at least 1 first-degree relative. A total of 396 first-degree relatives — 220 in a derivation cohort and 176 in a validation cohort — were enrolled in the study, and liver fibrosis was evaluated using magnetic resonance elastography and other noninvasive imaging modalities. The primary outcome was prevalence of advanced fibrosis in first-degree relatives.

**RESULTS:**

Prevalence of advanced fibrosis in first-degree relatives of probands with NAFLD with advanced fibrosis, NAFLD without advanced fibrosis, and non-NAFLD was 15.6%, 5.9%, and 1.3%, respectively (*P* = 0.002), in the derivation cohort, and 14.0%, 2.6%, and 1.3%, respectively (*P* = 0.004), in the validation cohort. In multivariable-adjusted logistic regression models, age of ≥50 years (adjusted OR [aOR]: 2.63, 95% CI 1.0–6.7), male sex (aOR: 3.79, 95% CI 1.6–9.2), diabetes mellitus (aOR: 3.37, 95% CI 1.3–9), and a first-degree relative with NAFLD with advanced fibrosis (aOR: 11.8, 95% CI 2.5–57) were significant predictors of presence of advanced fibrosis (all *P* < 0.05).

**CONCLUSION:**

First-degree relatives of probands with NAFLD with advanced fibrosis have significantly increased risk of advanced fibrosis. Routine screening should be done in the first-degree relatives of patients with advanced fibrosis.

**FUNDING:**

Supported by NCATS (5UL1TR001442), NIDDK (U01DK061734, U01DK130190, R01DK106419, R01DK121378, R01DK124318, P30DK120515, K23DK119460), NHLBI (P01HL147835), and NIAAA (U01AA029019); Academy of Finland grant 309263; the Novo Nordisk, EVO, and Sigrid Jusélius Foundations; and the Innovative Medicines Initiative 2 Joint Undertaking under grant agreement 777377. This Joint Undertaking receives support from the European Union’s Horizon 2020 research and innovation program and the EFPIA.

## Introduction

Nonalcoholic fatty liver disease (NAFLD) afflicts approximately one-fourth of the general population worldwide (1). Since a subset of patients with NAFLD progress to nonalcoholic steatohepatitis, hepatocellular carcinoma, and liver failure, NAFLD has emerged as an important health and economic burden (2). NAFLD cirrhosis has become one of the leading indications for liver transplantation (3).

Recent studies have demonstrated that advanced fibrosis defined as histological stage 3 and stage 4 fibrosis is the most important prognostic determinant of liver-related morbidity and mortality in patients with NAFLD (4–6). Although liver biopsy is the gold standard for the assessment of liver fibrosis, it has several limitations, including sampling variability and intra- and interobserver reproducibility (7). Therefore, noninvasive identification of advanced fibrosis among patients with NAFLD, who are at a higher risk of liver-related morbidity and mortality, is a major unmet need in clinical practice. Noninvasive imaging modalities for the assessment of liver fibrosis that assess liver stiffness, such as vibration-controlled transient elastography (VCTE) and magnetic resonance elastography (MRE), have been developed and are increasingly used in routine clinical practice (8, 9). However, given the high global burden of NAFLD and the limited availability of VCTE and MRE, it is impractical to assess for liver fibrosis in all patients even with noninvasive methods. Therefore, it is important to identify a subset of patients who are at a higher risk for advanced fibrosis using routine history and clinical risk stratification.

NAFLD is a complex metabolic disease with underlying genetic and environmental risk factors, and recent studies have demonstrated that NAFLD and NAFLD-related liver fibrosis are heritable (10–15). Therefore, NAFLD with advanced fibrosis may cluster within the same families.

The current American Gastroenterological Association and American Association for the Study of Liver Diseases guidelines do not recommend screening for advanced fibrosis among first-degree relatives of patients with cirrhosis/advanced fibrosis (16, 17). A previous pilot single-center study showed that first-degree relatives of probands with NAFLD cirrhosis have a higher risk of advanced fibrosis. These data need to be validated before clinical practice guidelines are changed to recommend routine screening in this high-risk population. Therefore, we aimed to validate the prevalence of advanced fibrosis among first-degree relatives of patients with advanced fibrosis due to NAFLD using 2 uniquely well-phenotyped independent cohorts derived from populations residing in the United States and Europe, respectively.

**Figure 1:**
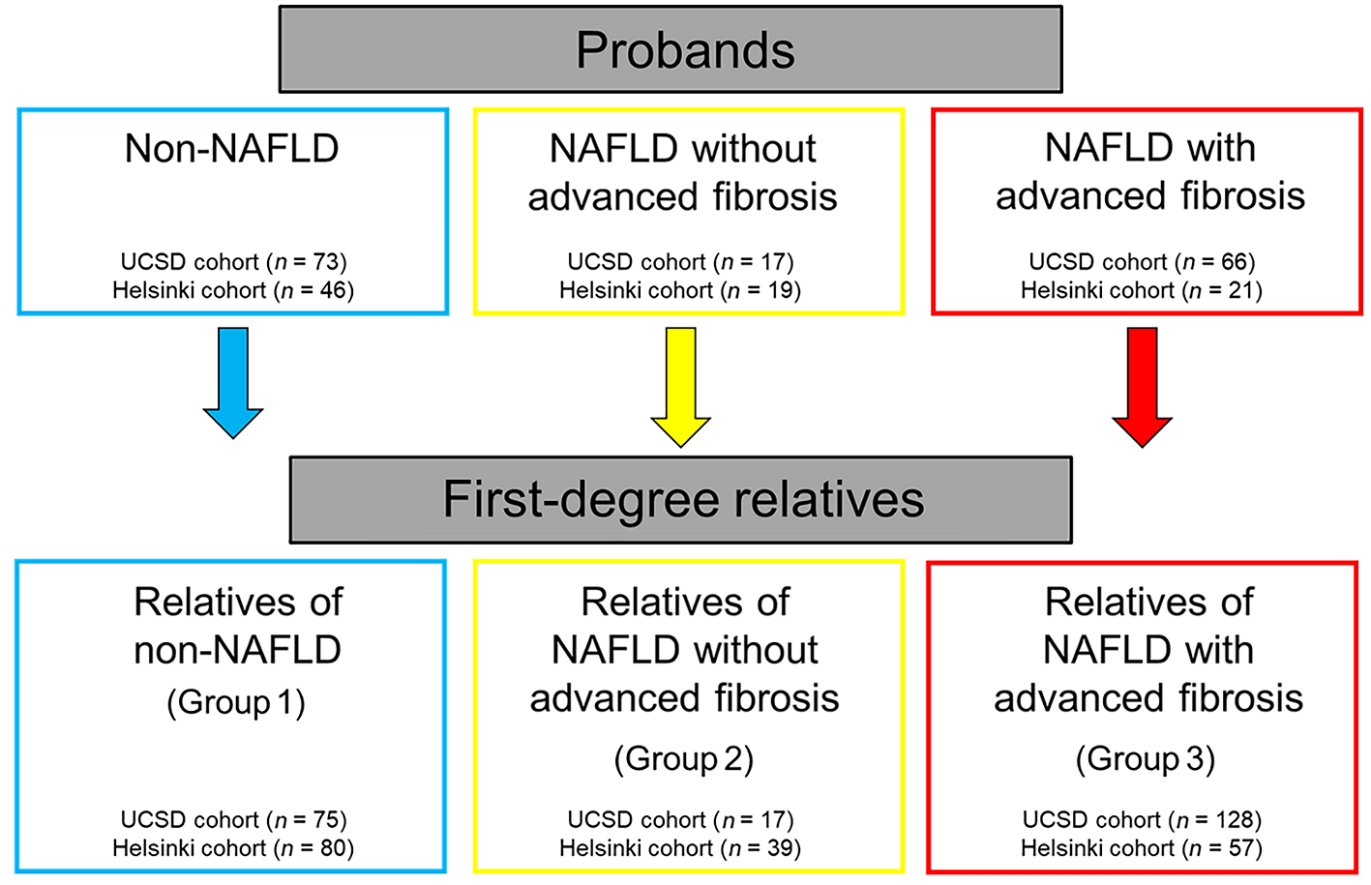
Study design.

We hypothesized that the prevalence of advanced fibrosis is higher in first-degree relatives of probands with NAFLD with advanced fibrosis than in first-degree relatives of probands with NAFLD without advanced fibrosis or non-NAFLD controls. Using a prospective cohort study design including a derivation cohort from the University of California San Diego (UCSD) and a validation cohort from the University of Helsinki, we aimed to determine the prevalence of advanced fibrosis among first-degree relatives of probands with NAFLD with advanced fibrosis versus first-degree relatives of probands without advanced fibrosis or non-NAFLD controls (Figure 1). Furthermore, we examined factors associated with advanced fibrosis in first-degree relatives.

## Results

### Characteristics of first-degree relatives.

A total of 396 first-degree relatives were enrolled in the study. The UCSD cohort comprised 220 first-degree relatives who were grouped into 3 subgroups stratified by their proband’s status: group 1, relatives of probands with non-NAFLD; group 2, relatives of probands with NAFLD without advanced fibrosis; and group 3, relatives of probands with NAFLD with advanced fibrosis. Similarly, the Helsinki cohort comprised 176 first-degree relatives: 80 in group 1, 39 in group 2, and 57 in group 3 (Table 1).

In the UCSD cohort, the median (interquartile range [IQR]) ages in group 1, group 2, and group 3 were 41 (23 to 60), 54 (48 to 58), and 46 (35 to 60) years, respectively. Approximately half of the UCSD cohort (50.9%, 112/220) had Hispanic ethnicity. In the Helsinki cohort, the median (IQR) ages in group 1, group 2, and group 3 were 48 (32 to 62), 50 (32 to 64), and 46 (34 to 61) years, respectively. All relatives were White in the Helsinki cohort. In both the UCSD and the Helsinki cohort, younger subjects were more likely to be enrolled in group 3 than in group 1 and group 2. The prevalence of diabetes mellitus (DM) was higher in groups 2 and 3 than in group 1.

### Prevalence of NAFLD and advanced fibrosis in first-degree relatives.

**Figure 2:**
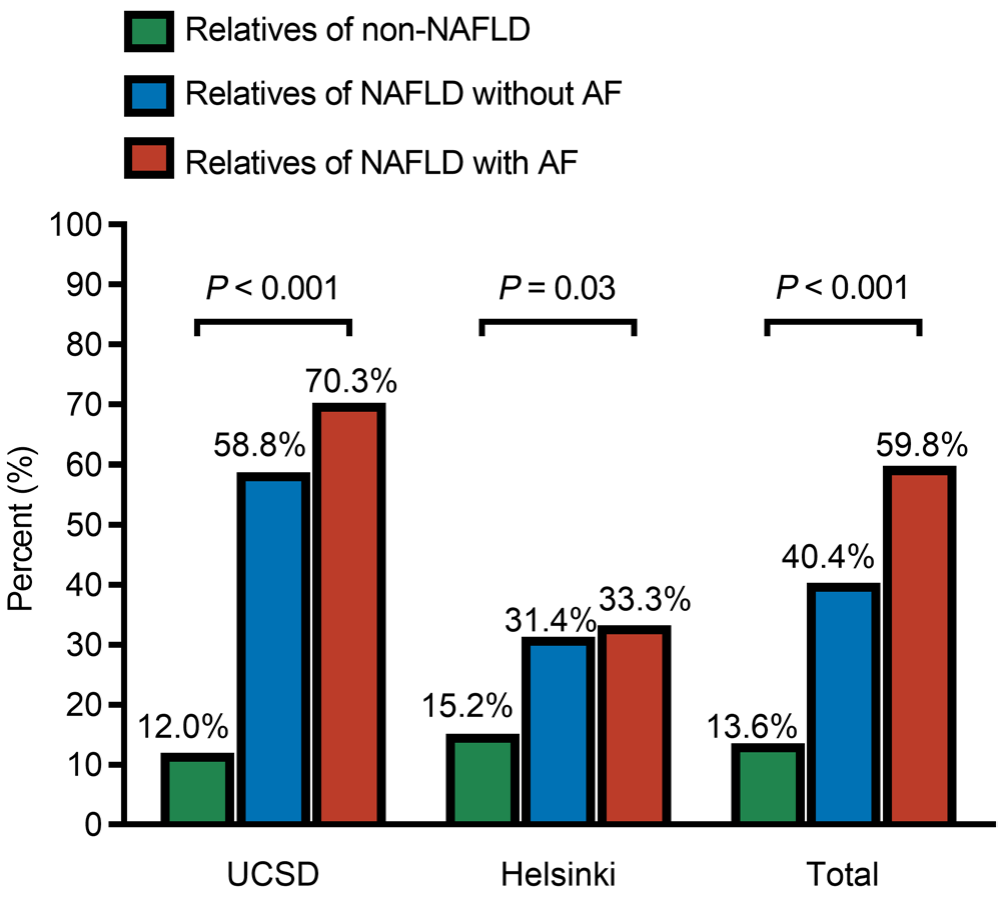
Prevalence of NAFLD in first-degree relatives. Group 1: Relatives of non-NAFLD controls. Group 2: Relatives of probands with NAFLD without advanced fibrosis (AF). Group 3: Relatives of probands with NAFLD with advanced fibrosis.

In the UCSD cohort, the prevalence of NAFLD in group 1, group 2, and group 3 was 12.0% (9/75), 58.8% (10/17), and 70.3% (90/128), respectively. As expected, the prevalence rate of NAFLD increased in a dose-dependent manner based on the NAFLD severity in the probands (*P* < 0.001; Figure 2). In the Helsinki cohort, the prevalence of NAFLD in group 1, group 2, and group 3 was 15.2% (12/79), 31.4% (11/35), and 33.3% (17/51), respectively, and it was higher in groups 2 and 3 than in group 1 (*P* = 0.03). In the combined cohort analysis, the prevalence of NAFLD in group 1, group 2, and group 3 was 13.6% (21/154), 40.4% (21/52), and 59.8% (107/179), respectively, and it increased with an increase in the severity of NAFLD in the probands (*P* < 0.001).

**Figure 3:**
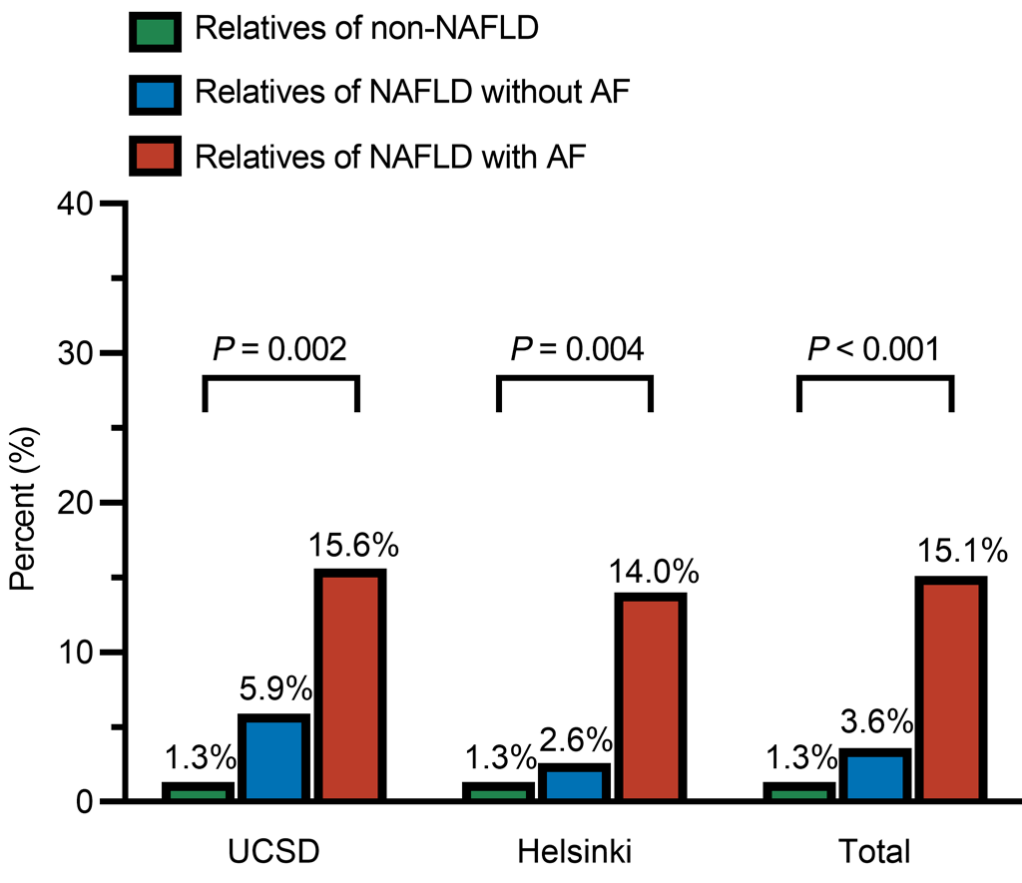
Prevalence of advanced fibrosis in first-degree relatives. Group 1: Relatives of non-NAFLD controls. Group 2: Relatives of probands with NAFLD without advanced fibrosis (AF). Group 3: Relatives of probands with NAFLD with advanced fibrosis.

Next, we investigated the prevalence of advanced fibrosis in the first-degree relatives. In the UCSD cohort, the prevalence of advanced fibrosis in group 1, group 2, and group 3 was 1.3% (1/75), 5.9% (1/17), and 15.6% (20/128), respectively. In comparison with groups 1 and 2, the prevalence rate of advanced fibrosis was statistically and clinically significantly higher in group 3 (*P* = 0.002; Figure 3). In the Helsinki cohort, the prevalence of advanced fibrosis in group 1, group 2, and group 3 was 1.3% (1/80), 2.6% (1/39), and 14.0% (8/57), respectively (*P* = 0.004). The results remained consistent and statistically significant, with a significantly higher prevalence rate in group 3 compared with groups 1 and 2, respectively. In the combined cohort analysis, the prevalence of advanced fibrosis in group 1, group 2, and group 3 was 1.3% (2/155), 3.6% (2/56), and 15.1% (28/185), respectively, and the prevalence rate of advanced fibrosis increased in a dose-dependent manner based on the severity of NAFLD in the probands (*P* < 0.001).

### Factors associated with advanced fibrosis in the first-degree relatives.

**Figure 4:**
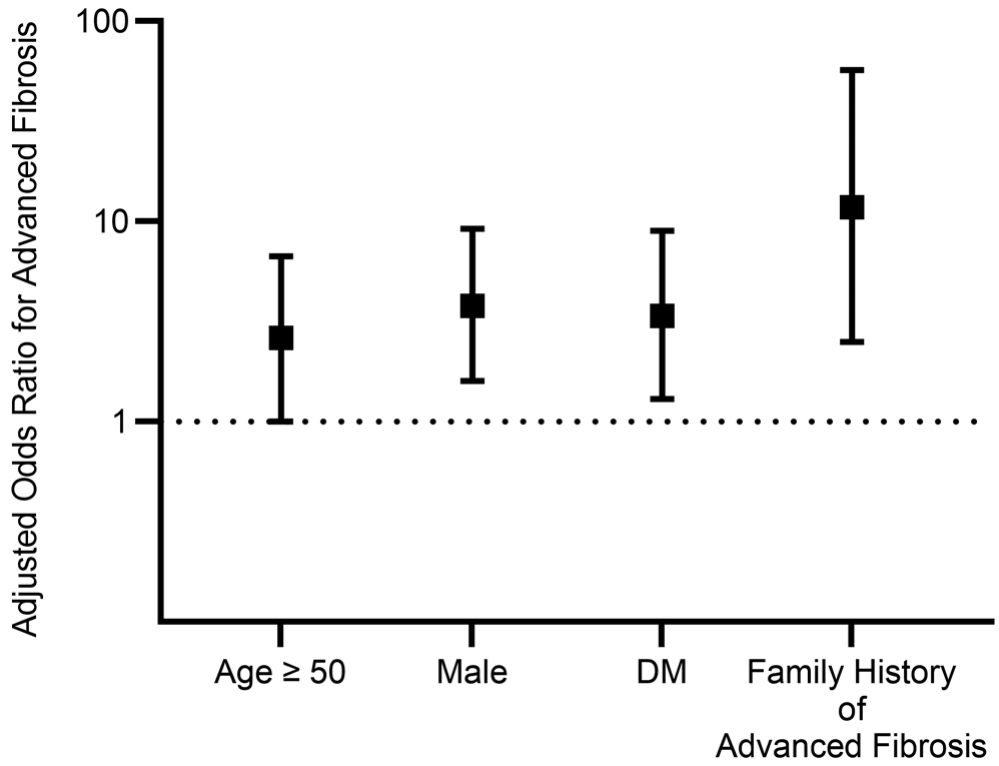
Odds ratio for advanced fibrosis. Age, sex, ethnicity, obesity, DM, dyslipidemia, hypertension, and family history of advanced fibrosis were adjusted in the multivariable analysis, and adjusted odds ratios of independent factors for advanced fibrosis are shown.

We then examined the factors associated with advanced fibrosis in first-degree relatives in the combined cohort (Table 2). In univariable analysis, proband status was a significant predictor of advanced fibrosis in the first-degree relatives. A first-degree relative of a proband with NAFLD with advanced fibrosis had 13.6 times (95% CI 3.2–58, *P* < 0.001) higher odds of having advanced fibrosis than a first-degree relative of a non-NAFLD control. Similarly, age of at least 50 years, ethnicity, obesity (BMI ≥30 kg/m^2^), DM, dyslipidemia, and hypertension were significant risk factors for advanced fibrosis in the first-degree relatives. In multivariable-adjusted logistic regression analyses, proband status of NAFLD with advanced fibrosis was a statistically significant and independent predictor of advanced fibrosis with a multivariable adjusted OR of 11.8 (95% CI 2.5–57, *P* = 0.002). The risk of advanced fibrosis in the first-degree relatives of probands with advanced fibrosis was independent of age ≥50 years (OR: 2.63, 95% CI 1.0–6.7, *P* = 0.04), male sex (OR: 3.79, 95% CI 1.6–9.2, *P* = 0.003), and DM (OR: 3.37, 95% CI 1.3–9.0, *P* = 0.02; Table 2 and Figure 4).

Furthermore, we conducted 2 sensitivity analyses to assess whether the findings were independent of DM status or age. Even after exclusion of relatives with DM, proband status of NAFLD with advanced fibrosis remained a clinically and statistically significant predictor of advanced fibrosis with an adjusted OR of 16.8 (95% CI 1.9–149.2, *P* = 0.01; Table 3). Similarly, after exclusion of relatives at least 50 years old, proband status of NAFLD with advanced fibrosis remained a clinically and statistically significant predictor of advanced fibrosis with an adjusted OR of 15.0 (95% CI 1.5–146.0, *P* = 0.02; Table 4).

## Discussion

### Main findings.

Using advanced imaging modalities to uniquely phenotype 2 geographically distinct independent study cohorts (one residing in Southern California and one residing in Helsinki, Finland), we provide clinical validation that the prevalence of advanced fibrosis among first-degree relatives of patients with advanced fibrosis due to NAFLD is approximately 15%. Furthermore, age of at least 50 years, male sex, DM, and proband status (NAFLD with advanced fibrosis) were independent predictors of advanced fibrosis due to NAFLD. We performed sensitivity analyses by excluding relatives with DM, and the results remained consistent. Therefore, the risk of advanced fibrosis among relatives of probands with advanced fibrosis is not mediated by DM status. This study provides important data regarding the prevalence of advanced fibrosis in first-degree relatives of probands with advanced fibrosis. These data suggest that a family history of advanced fibrosis may warrant further screening for liver fibrosis due to NAFLD among first-degree relatives of probands with advanced fibrosis. When patients with NAFLD-related advanced fibrosis are seen in liver clinics, their first-degree relatives should be counseled for risk of advanced fibrosis among family members, and they should be offered screening for advanced fibrosis with either MRE or VCTE or other modalities.

### In context with published literature.

Recent seminal studies have demonstrated that NAFLD and NAFLD-related liver fibrosis are heritable (10–15). In studies investigating the prevalence of NAFLD in offspring, the prevalence of NAFLD is higher in offspring with a parental history of NAFLD (10–12). Studies using a novel twin study design also demonstrated that the presence of NAFLD correlated between monozygotic twins but not between dizygotic twins (13). These results espouse the heritability of NAFLD.

Several genome-wide association studies revealed an association between NAFLD and single-nucleotide polymorphisms (SNPs) including *PNPLA3*, *TM6SF2*, and *GCKR* (18). These SNPs are associated with the accumulation of fat in liver and affect the development of NAFLD (19–21). These results also showed the association between genetic factors and NAFLD.

Importantly, liver fibrosis is the most important factor for prognosis in patients with NAFLD, and the heritability of NAFLD-related fibrosis warrants further investigation. The twin studies demonstrated that liver fibrosis and liver fat (the presence of NAFLD) have shared genetic effects and liver fibrosis also could be heritable (13–15). A study investigating the association between genetic risk and liver fibrosis by MRE demonstrated that the *PNPLA3* risk variant is associated with an increase in liver fibrosis (22). Therefore, not only the presence of NAFLD but also NAFLD-related fibrosis could be heritable, and we hypothesized that the prevalence of advanced fibrosis is higher in the first-degree relatives of probands with NAFLD with advanced fibrosis than in those of probands with NAFLD without advanced fibrosis or non-NAFLD.

In a previous proof-of-concept study including 39 first-degree relatives of probands with NAFLD with cirrhosis and 69 first-degree relatives of probands with non-NAFLD, Caussy and colleagues demonstrated that the prevalence of advanced fibrosis was higher in first-degree relatives of probands with NAFLD with cirrhosis than in those of probands with non-NAFLD (23). The prevalence of genetic risk variants associated with NAFLD and NAFLD-related fibrosis differs by ethnicity and region (1). Therefore, in order to validate the previous results of the proof-of-concept study, a validation study using diverse cohorts with a larger population was needed before a change in clinical practice guidelines could be implemented. In the present study, we investigated the prevalence of advanced fibrosis in first-degree relatives using 2 regionally independent cohorts with a total of 396 first-degree relatives. This study provides much-needed validation that the prevalence of advanced fibrosis among first-degree relatives of patients with advanced fibrosis due to NAFLD is approximately 15%. Using 2 uniquely well-phenotyped independent cohorts from the United States and Europe, this study provides key data to inform clinical practice guidelines.

In addition to genetic factors, NAFLD progression is associated with environmental factors (24). NAFLD is closely related to metabolic disorders including DM, dyslipidemia, and hypertension, and these factors are well-known risk factors for liver fibrosis in patients with NAFLD (24). Furthermore, aging is also a significant factor for liver fibrosis (25). In this study, we demonstrated that the proband status (NAFLD with advanced fibrosis) is a significant factor associated with advanced fibrosis in first-degree relatives independent of age, male sex, and DM. Furthermore, the odds ratio reflecting the degree of risk for advanced fibrosis was highest for the proband status for advanced fibrosis. Therefore, the proband status confers a higher susceptibility toward advanced fibrosis independent of age, male sex, and presence of DM. A family history of advanced fibrosis may be used as a screening tool for detecting subjects who are at a higher risk of advanced fibrosis in the general population.

### Strengths and limitations.

In this prospective study, all participants received systematic and standardized liver disease assessment, and other chronic liver diseases were excluded. Furthermore, all participants received a liver fibrosis assessment, primarily with MRE and a subset with ultrasound-based modalities through a standardized protocol. These modalities have high diagnostic accuracy for liver fibrosis and steatosis and are used in clinical trials (8, 19). The significant association between proband status and the prevalence of advanced fibrosis in first-degree relatives was confirmed in 2 geographically distinct cohorts. NAFLD progression, including fibrosis progression, is associated with menopausal status in women, and the prevalence of NAFLD and NAFLD-related fibrosis is higher in postmenopausal than in premenopausal women (26). Therefore, the effect of menopausal status on the prevalence of advanced fibrosis in relatives should be examined in a future study. This study includes mainly White and Hispanic participants, and future studies in other regions are needed to validate these findings. Furthermore, although genetic and environmental (lifestyle, cohabitation, exercise, diet, etc.) factors are associated with disease progression in NAFLD, we did not evaluate these factors in this study. Further studies are needed to investigate the role of genes, environment, and their interaction in the risk for advanced fibrosis among family member of patients with NAFLD. Since this study mainly included patients with advanced fibrosis, the prevalence of advanced fibrosis remains to be quantified and assessed in the non-NAFLD controls and in the general population.

### Future implications.

NAFLD patients are widely distributed in the general population, and effective screening for subjects who are at high risk for advanced fibrosis is an unmet clinical need. Recent American Gastroenterological Association (AGA) and American Association for the Study of Liver Diseases (AASLD) practice guidelines state that the prevalence of advanced fibrosis among family members of patients with NAFLD is unknown. Therefore, systematic screening of family members of patients with NAFLD is currently not recommended unless they themselves have risk factors such as DM (16, 17). However, here we demonstrate that the prevalence of advanced fibrosis among first-degree relatives of probands with advanced fibrosis is approximately 15%. Moreover, first-degree relatives of probands with NAFLD with advanced fibrosis have significantly higher odds for advanced fibrosis independent of age, male sex, and the presence of DM. Therefore, this study provides new data to justify systematic screening for advanced fibrosis based on family history of advanced fibrosis due to NAFLD. These data have important implications for clinical practice and upcoming AGA and AASLD practice guidelines. Further studies are needed to determine whether genetic testing may further modify this risk and whether it would be cost-effective to perform routine genetic testing in clinical practice (18, 20, 21). This study demonstrated that high-risk patients could be detected using routine history and systematic assessment of family history and could be offered targeted screening with either MRE or VCTE or other noninvasive modalities for the presence of advanced fibrosis in this population. Therefore, screening for advanced fibrosis in first-degree relatives of probands with NAFLD with advanced fibrosis may be useful and may potentially be cost-effective for detecting high-risk patients, and the approach used in this study provides a practical screening strategy.

In conclusion, first-degree relatives of probands with NAFLD with advanced fibrosis have significantly increased risk of advanced fibrosis. Routine screening for advanced fibrosis should be done in the first-degree relatives of patients with advanced fibrosis. These data have important implications for clinical practice.

## Methods

### Study design.

This prospective study included 2 geographically distinct cohorts of participants, one residing in Southern California, the UCSD (derivation) cohort, and the other residing in Finland, the Helsinki (validation) cohort. In the UCSD cohort, probands with NAFLD with advanced fibrosis (*n* = 66), NAFLD without advanced fibrosis (*n* = 17), or non-NAFLD (*n* = 73) were enrolled in the study along with their first-degree relatives. In NAFLD without advanced fibrosis and non-NAFLD, twin pairs were also included in the study. All subjects were recruited from December 2011 to July 2021 (Figure 1). In the Helsinki cohort, all probands (NAFLD with advanced fibrosis, *n* = 21; NAFLD without advanced fibrosis, *n* = 19; and non-NAFLD, *n* = 46) were enrolled in the study initially, and at least 1 first-degree relative was subsequently recruited in the study from November 2017 to March 2021. The baseline characteristics of probands are shown in Supplemental Table 1 (supplemental material available online with this article; https://doi.org/10.1172/JCI162513DS1). Relatives of probands with non-NAFLD, NAFLD without advanced fibrosis, and NAFLD with advanced fibrosis were defined as groups 1, 2, and 3, respectively. All subjects completed written informed consent prior to enrollment.

### Inclusion and exclusion criteria.

All probands with at least 1 first-degree relative enrolled in the study were included. Probands and their first-degree relatives were included if they were adults at least 18 years old in the UCSD cohort, and between 18 and 74 years old in the Helsinki cohort. All probands and their first-degree relatives in both cohorts underwent a standardized medical history, anthropometric measurements, physical examination, and biochemical testing, as well as assessment of liver fibrosis and steatosis, which was by MRI-based modalities in the majority of participants; ultrasound-based modalities were used in participants who were unable to schedule or unable to undergo MRI assessment. All probands and relatives in both cohorts were assessed for other liver diseases (e.g., alcohol-related liver disease, viral hepatitis, autoimmune hepatitis, and primary biliary cholangitis), and subjects with evidence of chronic liver disease other than NAFLD were excluded.

Exclusion criteria (for both probands and relatives) in both cohorts included any of the following: (a) significant alcohol consumption (defined as ≥14 drinks per week for men or ≥7 drinks per week for women) within the previous 2-year period; (b) underlying liver disease including hepatitis B, hepatitis C, hemochromatosis, Wilson’s disease, α_1_-antitrypsin deficiency, glycogen storage disease, autoimmune hepatitis, and cholestatic or vascular liver disease; (c) clinical or laboratory evidence of secondary causes or chronic conditions associated with hepatic steatosis including nutritional disorders and HIV infection; (d) use of steatogenic drugs such as amiodarone, glucocorticoids, methotrexate, l-asparaginase, and valproic acid; (e) major systemic illnesses; (f) pregnancy or attempting to become pregnant, or lactation.

### Definition of NAFLD and advanced fibrosis.

NAFLD was defined by either proton density fat fraction (PDFF) ≥ 5.0% (27), controlled attenuation parameter (CAP) ≥ 288 decibels per meter (dB/m) (28), or proton magnetic resonance spectroscopy (^1^H-MRS) ≥ 5.56% (29) in all subjects.

In probands, advanced fibrosis was defined by either history of liver transplantation due to NAFLD, the presence of ascites, hepatic encephalopathy, varices, MRE ≥ 3.63 kPa (30), VCTE ≥ 10 kPa (31), or histological fibrosis stage 3 to 4 by Nonalcoholic Steatohepatitis Clinical Research Network Histologic Scoring System (32). Based on these criteria, probands were stratified as having NAFLD with advanced fibrosis, NAFLD without advanced fibrosis, or non-NAFLD. None of the probands who were the non-NAFLD controls had advanced fibrosis and NAFLD.

In first-degree relatives, advanced fibrosis was defined by previously validated criteria using either MRE ≥ 3.63 kPa (30) or VCTE ≥ 10 kPa (31) or acoustic radiation force impulse (ARFI) ≥ 2.07 m/s (33) regardless of steatosis status because liver fat decreases as liver fibrosis increases to advanced fibrosis or cirrhosis, a phenomenon known as burned-out nonalcoholic steatohepatitis (34). In the UCSD cohort, liver fibrosis was assessed by either MRE (75.0%, 165/220), VCTE (12.3%, 27/220), or ARFI (12.7%, 28/220), and liver steatosis was assessed by PDFF (87.7%, 193/220) or CAP (12.3%, 27/220). In the Helsinki cohort, liver fibrosis was assessed by MRE (96.0%, 169/176) or VCTE (4.0%, 7/176), and liver steatosis was assessed by ^1^H-MRS (90.3%, 159/176) or CAP (3.4%, 6/176); the assessment for liver steatosis was not available in 11 relatives.

### Imaging assessment for fibrosis and steatosis.

Advanced magnetic resonance examinations including MRE and PDFF using a 3T research scanner (GE Signa EXCITE HDxt, GE Healthcare) at the UCSD Liver Imaging Group or MRE and ^1^H-MRS using a 1.5T research scanner (GE Signa HDxt, GE Healthcare) at SYNLAB were performed to assess liver fibrosis and steatosis. The details of the MRI protocol have been described previously (27, 35). Image analysts at each site were blinded to all clinical and biochemical data. PDFF ≥ 5.0% (27) or ^1^H-MRS ≥ 5.56% (29) was considered as NAFLD (the presence of steatosis). MRE ≥ 3.63 kPa was used for the definition of advanced fibrosis (30).

VCTE and/or CAP examinations were obtained by trained operators using the FibroScan 502 Touch model (Echosens) at UCSD and the FibroScan Mini 430 model (Echosens) at Helsinki according to previously described methods (36, 37). According to manufacturer protocol, all examinations were initiated with M probe, and XL probe was used only when prompted by the automatic probe selection tool. Image analysts at each site were blinded to all clinical and biochemical data. VCTE ≥ 10 kPa and CAP ≥ 288 dB/m were defined as advanced fibrosis and NAFLD, respectively (28, 31).

In the UCSD cohort, ARFI was also used for liver fibrosis assessment in first-degree relatives. ARFI was obtained by trained operators using the Acuson S2000 (Siemens) according to previously described methods (38). The image analysts were blinded to all clinical and biochemical data. ARFI ≥ 2.07 m/s was used for the definition of advanced fibrosis based on a previous meta-analysis (33).

### Definition of comorbidities.

DM was defined using the American Diabetes Association criteria of hemoglobin A_1c_ ≥ 6.5%, fasting glucose > 125 mg/dL, or drug treatment (39). Dyslipidemia was defined by fasting high-density lipoprotein cholesterol < 40 mg/dL in males or < 50 mg/dL in females, fasting triglyceride > 150 mg/dL, or drug treatment. Hypertension was defined by blood pressure reading > 130/85 mmHg, or drug treatment.

### Primary outcome and secondary outcome.

The primary outcome was the prevalence of advanced fibrosis in the first-degree relatives. The secondary outcome was the factors associated with advanced fibrosis in first-degree relatives.

### Statistics.

The prevalence of NAFLD and advanced fibrosis in first-degree relatives was compared between the 3 groups using Fisher’s exact test. A threshold of age for advanced fibrosis was determined by the receiver operating characteristic curve and the Youden index. Univariable and multivariable logistic regression analyses were performed for factors associated with advanced fibrosis. Age, sex, ethnicity, BMI, DM, dyslipidemia, and hypertension are known risk factors for advanced fibrosis in NAFLD, and these factors were selected a priori for multivariable-adjusted logistic regression analysis. Statistical significance was defined as *P* less than 0.05. All statistical analyses were performed using EZR (Saitama Medical Center, Jichi Medical University, Saitama, Japan) (40) and a graphical user interface for R (R Foundation for Statistical Computing).

### Study approval.

The research protocol was approved by the Office of IRB administration UCSD and the Ethics Committee of the Hospital District of Helsinki and Uusimaa (Helsinki, Finland).

## Author contributions

Rohit L designed the study. All authors obtained the clinical data. NT and NA analyzed the data. NT, NA, PKL, KP, HYJ, and Rohit L interpreted the clinical data and analyzed data. NT, NA, PKL, KP, HYJ, and Rohit L wrote the draft manuscript. NT, NA, PKL, KP, SRS, VA, YK, SD, AA, VS, MJW, HP, HG, VC, CH, SJL, Ria L, AB, LR, PETA, KN, HI, HYJ, and Rohit L contributed to the critical revision of the manuscript and read and approved the final manuscript. HYJ and Rohit L supervised the study. NT and NA are co–first authors. The order of the co–first authors was determined based on their efforts and contributions to the manuscript.

## Supplementary Material

Supplemental data

Trial reporting checklists

ICMJE disclosure forms

## Figures and Tables

**Table 4:**
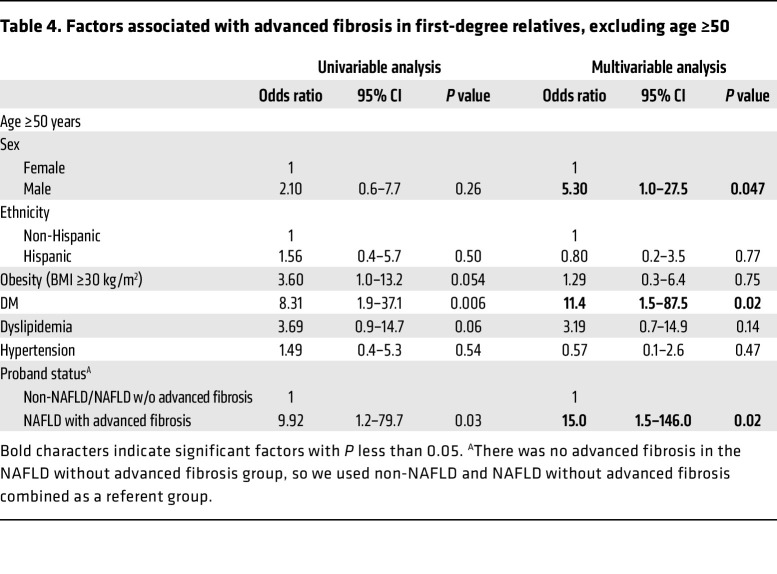
Factors associated with advanced fibrosis in first-degree relatives, excluding age ≥50

**Table 3:**
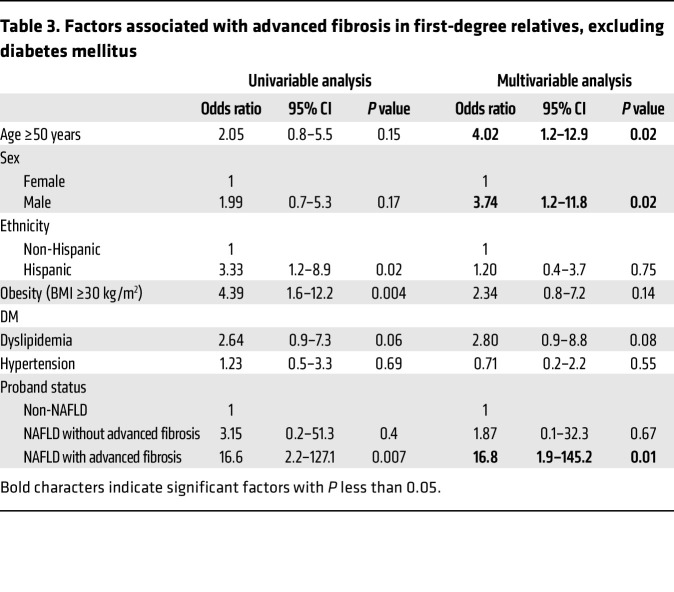
Factors associated with advanced fibrosis in first-degree relatives, excluding diabetes mellitus

**Table 2:**
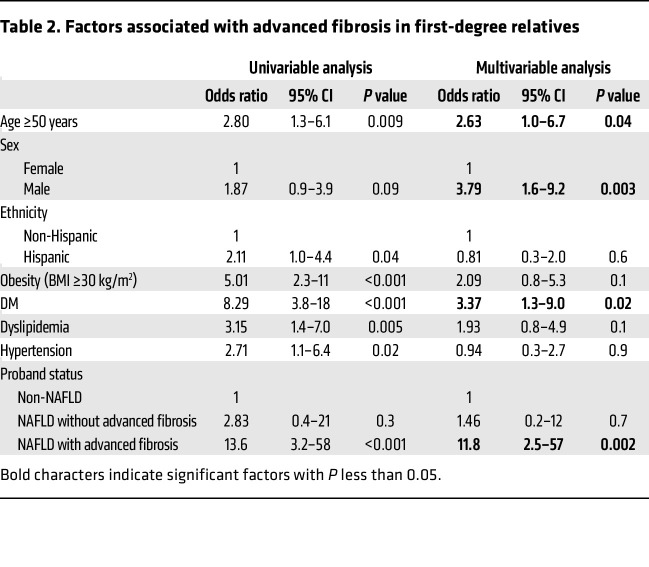
Factors associated with advanced fibrosis in first-degree relatives

**Table 1:**
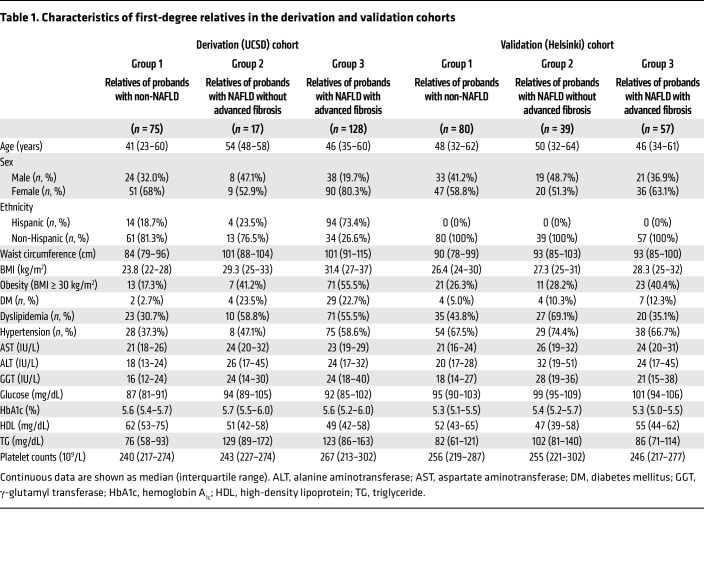
Characteristics of first-degree relatives in the derivation and validation cohorts

## Version 1 11/01/2022

Electronic publication
