# Supplementary material for: Risk of advanced fibrosis in first-degree relatives of patients with nonalcoholic fatty liver disease
Source: J Clin Invest. 2022 Nov 1;132(21):e162513. doi: 10.1172/JCI162513 (PMC9621132; doi:10.1172/JCI162513)
Supplement: Supplemental data [file jci-132-162513-s102.pdf]

Supplemental table 1. Characteristics of probands in the derivation and validation cohort

	UCSD (derivation) cohort			Helsinki (validation) cohort		
	Probands of non-NAFLD (n = 73)	Probands of NAFLD without advanced fibrosis (n = 17)	Probands of NAFLD with advanced fibrosis (n = 66)	Probands of non-NAFLD (n = 46)	Probands of NAFLD without advanced fibrosis (n = 19)	Probands of NAFLD with advanced fibrosis (n = 21)
Age (years)	54 (27–62)	57 (48–62)	63 (57–70)	55 (42–61)	56 (51–62)	65 (61–67)
Gender (female, n, %)	57 (78.1%)	9 (52.9%)	49 (74.2%)	35 (76.1%)	13 (68.4%)	9 (42.9%)
Race/ethnicity						
Hispanic (n, %)	13 (17.8%)	4 (23.5%)	42 (63.6%)	0 (0%)	0 (0%)	0 (0%)
Non-Hispanic (n, %)	60 (82.2%)	13 (76.5%)	24 (36.4%)	46 (100%)	19 (100%)	21 (100%)
Waist circumference (cm)	87 (80–96)	102 (95–114)	107 (97–115)	88 (79–98)	106 (93–113)	106 (100–117)
BMI (kg/m^2)	24.6 (22–28)	30.4 (29–37)	30.8 (37–36)	25.8 (23–29)	32.6 (28–34)	31.4 (29–36)
DM (n, %)	3 (4.1%)	8 (47.1%)	47 (71.2%)	5 (10.9%)	4 (21.1%)	18 (85.7%)
Dyslipidemia (n, %)	27 (37.0%)	13 (76.5%)	45 (68.2%)	19 (41.3%)	14 (73.7%)	17 (81.0%)
Hypertension (n, %)	26 (35.6%)	15 (88.2%)	54 (81.8%)	31 (67.4%)	16 (84.2%)	21 (100%)
AST (IU/L)	21 (18–26)	21 (17–24)	42 (34–54)	20 (16–25)	30 (20–54)	41 (26–54)
ALT (IU/L)	17 (13–25)	22 (17–31)	35 (26–48)	21 (17–29)	47 (28–92)	39 (25–55)
GGT (IU/L)	17 (12–23)	26 (17–35)	72 (45–133)	18 (14–24)	26 (20–64)	58 (30–125)
Glucose (mg/dl)	89 (83–93)	101 (90–113)	111 (95–147)	94 (89–101)	104 (99–113)	149 (119–172)
HbA1c (%)	5.6 (5.5–5.9)	6.1 (5.5–7.2)	6.3 (5.6–7.2)	5.3 (5.1–5.4)	5.6 (5.4–6.0)	7.2 (6.4–7.6)
HDL (mg/dl)	64 (52–76)	48 (39–51)	44 (36–54)	60 (50–69)	44 (38–57)	37 (34–48)
TG (mg/dl)	72 (60–100)	120 (88–193)	111 (91–148)	77 (55–91)	139 (83–158)	143 (107–215)
Platelet counts (10^9/L)	242 (213–292)	275 (246–325)	150 (96–202)	253 (203–281)	287 (242–304)	178 (145–206)

Continuous data are shown in median (interquartile range).

NAFLD, nonalcoholic fatty liver disease; BMI, body mass index; DM, diabetes mellitus; AST, aspartate aminotransferase; ALT, alanine aminotransferase; GGT, gamma glutamyl transferase; HbA1c, hemoglobin A1c, HDL, high-density lipoprotein; TG, triglyceride; UCSD, University of California San Diego
